# Supplementary material for: Trends of Mycobacterium tuberculosis and rifampicin resistance at the Ho Teaching Hospital in Ghana
Source: PLoS One. 2024 Jun 10;19(6):e0305161. doi: 10.1371/journal.pone.0305161 (PMC11164350; doi:10.1371/journal.pone.0305161)

PLOS ONE

Detection of Mycobacterium tuberculosis and Rifampicin Resistance at the Ho Teaching Hospital of Ghana

--Manuscript Draft--

**Manuscript Number:**	PONE-D-23-36410
**Article Type:**	Research Article
**Full Title:**	Detection of Mycobacterium tuberculosis and Rifampicin Resistance at the Ho Teaching Hospital ~~of~~ Ghana
**Short Title:**	Mycobacterium tuberculosis and Rifampicin Resistance

**Corresponding Author:**	John Gameli Deku<br>University of Health and Allied Sciences<br>Ho, GHANA

**Keywords:**	tuberculosis; Rifampicin-resistant; Ho Teaching Hospital

Abstract:

Background

Tuberculosis remains a major public health threat worldwide, causing significant morbidity and mortality, particularly in low- and middle-income countries. In recent years, efforts to combat tuberculosis have focused on strengthening healthcare systems and increasing access to diagnostics and treatment services. However, the success of these efforts depends on understanding the burden and geographical distribution of tuberculosis and the treatment options available. The aim of this study was to determine the burden and Rifampicin susceptibility of Mycobacterium tuberculosis ~~over a six-year period.~~

Methodology

A hospital-based retrospective cross-sectional study was conducted at the Ho Teaching Hospital, Ho, Ghana. Study data included archived results on tuberculosis testing using GeneXpert from 2016-2021. Archived data on tuberculosis testing was collected and entered using Microsoft Excel 2019. IBM SPSS (v26) was used for statistical analysis of the prevalence of tuberculosis. P-value <0.05 was considered statistically significant.

Results

The study encompassed 5128 presumptive tuberculosis cases from 2016 to 2021, revealing an overall prevalence of 10.76%. Males exhibited a significantly higher tuberculosis prevalence (14.20%) compared to females (7.48%), with a 2:1 male-to-female ratio. The burden of tuberculosis varied significantly across age groups, with those aged 30-45 years and 46-60 years facing twice the risk compared to those under 15 years. Rainy seasons correlated with heightened tuberculosis occurrences (12.12%) compared to the dry seasons (8.84%). Rifampicin-resistant tuberculosis was prevalent at 3.45%, slightly higher in females, particularly in the 45-59 age group (5.97%). Notably, tuberculosis prevalence exhibited fluctuations, peaking in 2016 (17.1%) and 2020 (11.5%), with a trough in 2019 (4.6%). Gender-based variations were consistent with this trend, highlighting the need for targeted interventions to address the complex dynamics of tuberculosis prevalence.

Conclusion

The overall prevalence of the infection is high, considering its infectious nature. This calls for concerted efforts in curbing the spread of the infection.

Order of Authors:

John Gameli Deku

Enoch Aninagyei

Israel Bedzina

Powered by Editorial Manager® and ProduXion Manager® from Aries Systems Corporation

	Gameli Nudo
	Emmanuel Ativi
	Prosper Mensah
	Solomon Wireko
	Aaron Osei-Tutu
	Emmanuel Duker
	Innocent Afeke
**Additional Information:**	
**Question**	**Response**
**Financial Disclosure**<br><br>Enter a financial disclosure statement that describes the sources of funding for the work included in this submission. Review the submission guidelines for detailed requirements. View published research articles from *PLOS ONE* for specific examples.<br><br>This statement is required for submission and **will appear in the published article** if the submission is accepted. Please make sure it is accurate.<br><br>**Funded studies**<br>Enter a statement with the following details:<br>• Initials of the authors who received each award<br>• Grant numbers awarded to each author<br>• The full name of each funder<br>• URL of each funder website<br>• Did the sponsors or funders play any role in the study design, data collection and analysis, decision to publish, or preparation of the manuscript?<br><br>Did you receive funding for this work?	The author(s) received no specific funding for this work.
**Competing Interests**<br><br>Use the instructions below to enter a competing interest statement for this submission. On behalf of all authors, disclose any competing interests that could be perceived to bias this work—acknowledging all financial support and any other relevant financial or non-	The authors declared no conflict of interest

financial competing interests.<br><br>This statement is required for submission and **will appear in the published article** if the submission is accepted. Please make sure it is accurate and that any funding sources listed in your Funding Information later in the submission form are also declared in your Financial Disclosure statement.<br><br>View published research articles from *PLOS ONE* for specific examples.<br><br>**NO authors have competing interests**<br><br>Enter: *The authors have declared that no competing interests exist.*<br><br>**Authors with competing interests**<br><br>Enter competing interest details beginning with this statement:<br><br>*I have read the journal's policy and the authors of this manuscript have the following competing interests: [insert competing interests here]*<br><br>* typeset	
**Ethics Statement**<br><br>Enter an ethics statement for this submission. This statement is required if the study involved:<br><br>• Human participants<br>• Human specimens or tissue<br>• Vertebrate animals or cephalopods<br>• Vertebrate embryos or tissues<br>• Field research<br><br>Write "N/A" if the submission does not require an ethics statement.<br><br>General guidance is provided below.<br>Consult the submission guidelines for	Ethical consideration<br>Ethical approval with reference number UHAS-REC A.1 [139] 22-23 was obtained from the University of Health and Allied Sciences Research and Ethics Committee. In addition, written permission was sought from the management of the Ho Teaching Hospital for the use of data generated at the microbiology unit at the facility for the study. Informed consent was not obtained from the participants since the study was a retrospective one. All archived data for the study was kept undisclosed and used for the study only by keeping them on a password protected computer accessible to only the principal investigator.

detailed instructions. **Make sure that all information entered here is included in the Methods section of the manuscript.**<br><br>**Format for specific study types**<br><br>**Human Subject Research (involving human participants and/or tissue)**<br>• Give the name of the institutional review board or ethics committee that approved the study<br>• Include the approval number and/or a statement indicating approval of this research<br>• Indicate the form of consent obtained (written/oral) or the reason that consent was not obtained (e.g. the data were analyzed anonymously)<br><br>**Animal Research (involving vertebrate animals, embryos or tissues)**<br>• Provide the name of the Institutional Animal Care and Use Committee (IACUC) or other relevant ethics board that reviewed the study protocol, and indicate whether they approved this research or granted a formal waiver of ethical approval<br>• Include an approval number if one was obtained<br>• If the study involved *non-human primates*, add *additional details* about animal welfare and steps taken to ameliorate suffering<br>• If anesthesia, euthanasia, or any kind of animal sacrifice is part of the study, include briefly which substances and/or methods were applied<br><br>**Field Research**<br><br>Include the following details if this study involves the collection of plant, animal, or other materials from a natural setting:<br>• Field permit number<br>• Name of the institution or relevant body that granted permission	
**Data Availability**<br><br>Authors are required to make all data underlying the findings described fully available, without restriction, and from the time of publication. PLOS allows rare	Yes - all data are fully available without restriction

exceptions to address legal and ethical concerns. See the PLOS Data Policy and FAQ for detailed information.<br><br>A Data Availability Statement describing where the data can be found is required at submission. Your answers to this question constitute the Data Availability Statement and **will be published in the article**, if accepted.<br><br>**Important:** Stating 'data available on request from the author' is not sufficient. If your data are only available upon request, select 'No' for the first question and explain your exceptional situation in the text box.<br><br>Do the authors confirm that all data underlying the findings described in their manuscript are fully available without restriction?	
**Describe where the data may be found in full sentences. If you are copying our sample text, replace any instances of XXX with the appropriate details.**<br><br>• If the data are **held or will be held in a public repository**, include URLs, accession numbers or DOIs. If this information will only be available after acceptance, indicate this by ticking the box below. For example: *All XXX files are available from the XXX database (accession number(s) XXX, XXX.*).<br>• If the data are all contained **within the manuscript and/or Supporting Information files**, enter the following: *All relevant data are within the manuscript and its Supporting Information files.*<br>• If neither of these applies but you are able to provide **details of access elsewhere**, with or without limitations, please do so. For example:<br><br>*Data cannot be shared publicly because of [XXX]. Data are available from the XXX Institutional Data Access / Ethics Committee (contact via XXX) for researchers who meet the criteria for*	All relevant data will be uploaded as a supporting file if the manuscript is accepted for publication

access to confidential data.<br><br>*The data underlying the results presented in the study are available from (include the name of the third party and contact information or URL).*<br>• This text is appropriate if the data are owned by a third party and authors do not have permission to share the data.<br><br>* typeset	
Additional data availability information:	

Powered by Editorial Manager® and ProduXion Manager® from Aries Systems Corporation

Manuscript

Click here to access/download;Manuscript;TB MANUSCRIPT 2023_1.docx

Detection of *Mycobacterium tuberculosis* and Rifampicin Resistance at the Ho Teaching Hospital of Ghana.

John Gameli Deku[1]*, Enoch Aninagyei[2], Israel Bedzina[3], Gameli Nudo[4], Ativi Emmanuel[4], Prosper Mensah[4], Solomon Wireko[5], Osei-Tutu Aaron[1], Duker Emmanuel[1] and Innocent Afeke[1].

[1]*Department of Medical Laboratory Sciences, School of Allied Health Sciences, University of Health and Allied Sciences, Ho, Ghana.*

[2]*Department of Biomedical Sciences, School of Basic and Biomedical Sciences, University of Health and Allied Sciences, Ho, Ghana.*

[3]*Reinbee Medical Laboratory and Wellness Center, Ho*

[4]*Laboratory Department, Ho Teaching Hospital, Ho, Ghana.*

[5]*Department of Laboratory Technology, Kumasi Technical University, Kumasi, Ghana.*

Emails:

JGD*: sssdeku@gmail.com

EA: eaninagyei@uhas.edu.gh

IB: israelbedzina@gmail.com

GN: gamelinudo@gmail.com

AE: ativiemmanuel@gmail.com

PM: prosmens@yahoo.co.uk

SW: excellence521@gmail.com

O-TA: Oseitutuaaron88@gmail.com

DE: kwophinanaduker@gmail.com

IA: iafeke@uhas.edu.gh

*Corresponding author

E mail: sssdeku@gmail.com

Abstract

Background

Tuberculosis remains a major public health threat worldwide, causing significant morbidity and mortality, particularly in low- and middle-income countries. In recent years, efforts to combat tuberculosis have focused on strengthening healthcare systems and increasing access to diagnostics and treatment services. However, the success of these efforts depends on understanding the burden and geographical distribution of tuberculosis and the treatment options available. The aim of this study was to determine the burden and Rifampicin susceptibility of ~~Mycobacterium tuberculosis~~ over a six-year period.

Methodology

A hospital-based retrospective cross-sectional study was conducted at the Ho Teaching Hospital, Ho, Ghana. Study data included archived results on tuberculosis testing using GeneXpert from 2016-2021. Archived data on tuberculosis testing was collected and entered using Microsoft Excel 2019. IBM SPSS (v26) was used for statistical analysis of the prevalence of tuberculosis. P-value <0.05 was considered statistically significant.

Results

The study encompassed 5128 presumptive tuberculosis cases from 2016 to 2021, revealing an overall prevalence of 10.76%. Males exhibited a significantly higher tuberculosis prevalence (14.20%) compared to females (7.48%), with a 2:1 male-to-female ratio. The burden of tuberculosis varied significantly across age groups, with those aged 30-45 years and 46-60 years facing twice the risk compared to those under 15 years. Rainy seasons correlated with heightened

tuberculosis occurrences (12.12%) compared to the dry seasons (8.84%). Rifampicin-resistant tuberculosis was prevalent at 3.45%, slightly higher in females, particularly in the 45-59 age group (5.97%). Notably, tuberculosis prevalence exhibited fluctuations, peaking in 2016 (17.1%) and 2020 (11.5%), with a trough in 2019 (4.6%). Gender-based variations were consistent with this trend, highlighting the need for targeted interventions to address the complex dynamics of tuberculosis prevalence.

Conclusion

The overall prevalence of the infection is high, considering its infectious nature. This calls for concerted efforts in curbing the spread of the infection.

Keywords: Tuberculosis, Rifampicin-resistant, Ho Teaching Hospital

1.0 Introduction

Tuberculosis, which is caused by bacteria of the *Mycobacterium tuberculosis (M. tuberculosis* complex), is one of the oldest diseases known to affect humans and a major cause of death worldwide [1]. *Mycobacterium africanum* (*M. africanum), Mycobacterium canetti (M. canetti), Mycobacterium caprae (M. caprae), Mycobacterium microti (M. microti),* and *Mycobacterium pinnipedii (M. pinnipedii)* are responsible for a negligible portion of human cases. People with pulmonary tuberculosis emit aerosolized germs, especially when coughing, which causes the disease to spread via the air [2].

A sustained immunological response to *M. tuberculosis* antigen stimulation is known as latent tuberculosis infection (LTBI). It is defined as the absence of clinically apparent active tuberculosis [3]. There is not a perfect test for LTBI. The WHO recommendations on LTBI take into account the possibility that a particular risk group will develop active tuberculosis disease, the epidemiology and burden of tuberculosis, the accessibility of resources, and the prospect of a significant negative impact on public health [3]. The environment, the host, and the pathogen interact to cause the development of tuberculosis disease. HIV coinfection, immunodeficiency, diabetes mellitus, crowded living conditions, malnutrition, and overall poverty are established risk factors [4].

A third of the globe is thought to be infected with *M. tuberculosis* but is asymptomatic, which is described as having latent tuberculosis, despite the fact that 9 million new cases of active tuberculosis are ~~being~~ recorded each year [4]. World Health Organization estimates that out of the more than 9 million persons who contract tuberculosis each year, more than 3 million do not receive a formal diagnosis, course of treatment, or registration by national tuberculosis programs

[5]. The issue gets worse with the emergence of multi-drug resistance tuberculosis. These "missed" million tuberculosis patients represent a failure of global public health as a whole [5]. Tuberculosis prevalence surveys are essential to accurately assess the prevalence and trajectory of tuberculosis in the general population, particularly in nations with high tuberculosis burdens, poor case detection rates, and underdeveloped reporting systems [6].

Policy makers can use the data from tuberculosis prevalence surveys to enhance reporting systems, as well as tuberculosis diagnosis and treatment programmes. To curb the spread of tuberculosis and lessen its impact, early diagnosis and treatment are essential. However, conducting routine tuberculosis prevalence surveys is expensive and logistically challenging [6]. The resurgence of tuberculosis as a global worry was caused by the lack of an effective vaccine, the lengthy and expensive drug regimens, the limited diagnostic tools available in endemic countries, and the dismantling in several nations of the health systems and control measures that so successfully contributed to control tuberculosis throughout most of the 20th century [7].

Although there has been progress, over the next three years, funding for research, care, and prevention needs to treble annually. With an emphasis on access to care and preventive treatment and a drive towards growing use of molecular tests to biologically confirm the condition, financial allowances for the significant expenses on patients and their contacts need to be expanded (Harding, 2020). There is scarcity of data on rifampicin-resistant tuberculosis. This study determined the prevalence of tuberculosis and their rifampicin resistance over a six-year period (2016-2021) at the Ho Teaching Hospital of Ghana.

2.0 Materials and Methods

2.1 Study design

The study was a hospital-based retrospective study which analysed secondary data collected on tuberculosis from the Microbiology Laboratory Unit of the Ho Teaching Hospital (HTH). Data analysed in this publication was archived from 2016-2021.

2.2 Study site

The study was conducted at the microbiology laboratory unit of the HTH **(Figure 1),** located in Ho Municipality, Volta Region. The microbiology unit of the HTH laboratory processes urine, stool, sputum, and other body site fluids for infectious pathogens. Ho is the capital city of the Volta Region of Ghana. The city lies between Mount Adaklu and Mount Galenukui. The population of Ho municipality is approximately 177,281, representing 8.4 percent of the region's total population according to the 2010 Population and Housing Census. Females constituted 52.7%, and males represented 43%. Approximately 62% of the population resides in urban localities [8]. The Municipality shares boundaries with Adaklu and Agotime - Ziope districts to the south, Ho west district to the north and west and the Republic of Togo to the east. Its total land area is 2361 square kilometres (912 sq. km), thus representing 1.5% of the region's total land area. HTH has coordinates 6.60126^{o}N 0.48404^{O}E The HTH is the main referral facility in the Volta region. It has over 100,000 patients and a bed capacity of 340.

Figure1. Location of Ho Teaching Hospital in Ho municipality

2.3 Study participants

The study participants included records of tuberculosis testing performed and archived between 2016 and 2021 at the Ho Teaching Hospital.

2.4 GeneXpert assay

Sputum samples with sufficient volume were tested with Xpert *Mycobacterium tuberculosis*/Rifampicin (MTB/RIF) Version 5.0 assay on the GeneXpert platform (Cepheid, USA) to detect the presence of *Mycobacterium tuberculosis* and rifampicin resistance, as per the manufacturer's instructions.

2.5 Data collection

Archived data from the hospital were collected from the hospital microbiology unit and later entered Microsoft Excel and prepared for analysis. The archived records were accessed between October 3, 2022, and October 22, 2022.

2.6 Data analysis

Data collected with Microsoft Excel 2019 were exported into IBM Statistical Package for the Social Sciences version 26.0 (SPSS v.26) later for statistical analysis. Descriptive statistics was done to analyze participants demographics and trends in TB prevalence over the period of study. A *P value* <0.05 was considered to indicate statistical significance.

2.7 Ethical consideration

Ethical approval with reference number UHAS-REC A.1 [139] 22-23 was obtained from the University of Health and Allied Sciences Research and Ethics Committee. In addition, written permission was sought from the management of the Ho Teaching Hospital for the use of data

generated at the microbiology unit at the facility for the study. Informed consent was not obtained from the participants since the study was a retrospective one. No information that could be linked to the participants was retrieved. All archived data for the study was kept undisclosed and used for the study only by keeping them on a password protected computer accessible to only the principal investigator.

3.0 Results

A total of 5128 presumptive tuberculosis cases were received for laboratory confirmation within the period between 2016 and 2021. Out of this, 2507 (48.89%) were males and the remaining 2621 (51.11%) were females. The overall prevalence of laboratory confirmed tuberculosis was 10.76% among the suspected cases. The prevalence of laboratory confirmed tuberculosis was significantly higher among males (14.20%) than their female counterpart (7.48%) with male to female ration of 2:1 ($p<0.001$). After adjusting for confounding variables males were twice more likely to be burdened with tuberculosis than their female counterparts (aOR2.07 (1.72-2.49). The burden of laboratory confirmed tuberculosis varied significantly across various age classes ($p < 0.001$) with persons below 15 years of age being the least burden. The highest risk of tuberculosis was highest among the middle aged [30-45 years (13.35%)]. The age groups 30 -45 years, and 46 – 60 years were twice more likely to have TB compared to those under 15 years [(aOR =2.74, 95%CI: 1.74 – 4.31, $p<0.01$) and (aOR =2.58, 95%CI: 1.64 – 34.07, $p<0.01$) respectively]. Also, climatic seasons were significantly associated with the occurrence of tuberculosis infection ($p<0.01$). Cases of tuberculosis were more frequently identified in the rainy season [365(12.12%), aOR=1.30, $p<0.01$] than in the dry season [187(8.84%)].

Table 1 Prevalence and factors of Tuberculosis among suspected cases at the Ho Teaching Hospital, 2016 to 2021

Characteristics	Total	MTB Detected	P-value	cOR (95% CI)	P-value	aOR (95% CI)	P-value
**Overall**	**5128**	**552(10.76)**					
**Age Group**							
<15 years	385	23(5.97)	<0.001	1		1	
15 - 29 years	907	86(9.48)		1.65(1.02 - 2.65)	0.040	1.92 (1.19-3.11)	**0.008**
30 - 44 years	1326	177(13.35)		2.42(1.55 - 3.80)	<0.001	2.74 (1.74-4.31)	**<0.001**
45 - 59 years	1292	167(12.93)		2.34(1.49 - 3.67)	<0.001	2.58 (1.64-4.07)	**<0.001**
>/= 60 years	1218	99(8.13)		1.39(0.87 - 2.23)	0.166	1.53 (0.95-2.45)	0.079
**Gender**							
Female	2621	196(7.48)	<0.001	1		1	
Male	2507	356(14.20)		2.05(1.70 - 2.46)	<0.001	2.07 (1.72-2.49)	**<0.001**
**YEAR for TB infection**							
2016	252	43(17.06)	<0.001	1		1	
2017	1158	144(12.44)		0.69(0.48 - 1.00)	0.051	0.71 (0.49-1.04)	0.080
2018	1444	139(9.63)		0.52(0.36 - 0.75)	0.001	0.55 (0.38-0.80)	**0.002**
2019	241	11(4.56)		0.23(0.12 - 0.46)	<0.001	0.27 (0.13-0.55)	**<0.001**
2020	1019	117(11.48)		0.63(0.43 - 0.92)	0.018	0.66 (0.45-0.97)	**0.036**
2021	1014	98(9.66)		0.52(0.35 - 0.77)	0.001	0.56 (0.37-0.83)	**0.004**
**Season**							
Dry Season	2116	187(8.84)	<0.001	1		1	
Rainy Season	3012	365(12.12)		1.42(1.18 - 1.71)	<0.001	1.30 (1.07-1.58)	**0.008**
**Quarter of the Year**							
First Quarter	1244	132(10.61)	<0.001	1			
Second Quarter	1002	122(12.18)		1.17(0.90 - 1.52)	0.245		
Third Quarter	1193	156(13.08)		1.27(0.99 - 1.62)	0.060		
Fourth Quarter	1689	142(8.41)		0.77(0.60 - 0.99)	0.043		

The overall prevalence of newly diagnosed rifampicin-resistant Tuberculosis (RR-TB) was 3.45%. The prevalence of RR-TB was comparably higher among females than males (4.00% vrs 3.16% respectively, p=0.395). Similarly, comparable prevalence of RR-TB was observed among the various age classes with patient 45 – 59 years having the highest burden of resistance with a prevalence of 5.97% (P=0.627). None of the patients below 15 years was diagnosed with RR-TB. Furthermore, the burden of newly diagnosed RR-TB varied significantly across the period under review (p=0.038). There was an oscillating trend in the prevalence of RR-TB across the period from 2016 (4.65%) through 2017 (2.80%), 2018 (4.32%), 2019 (0.00%) and 2020 (5.88%) to 2021 (1.43%). However seasonal variations were not associated with the occurrence of rifampicin resistance among the newly diagnosed tuberculosis cases (p=0.738).

Table 2 Prevalence and risk factors of newly diagnosed rifampicin resistant tuberculosis among suspected cases at the Ho Teaching Hospital between 2016 and 2021

Risk factor	Total	Susceptible	Indeterminate	Resistant	P-value
Overall	435	413(94.94)	7(1.61)	15(3.45)	
**Age Group**					
<15 years	19	19(100.00)	0(0.00)	0(0.00)	0.627
15 - 29 years	73	71(97.26)	1(1.37)	1(1.37)	
30 - 44 years	128	121(94.53)	2(1.56)	5(3.91)	
45 - 59 years	134	124(92.54)	2(1.49)	8(5.97)	
>/= 60 years	81	78(96.30)	2(2.47)	1(1.23)	
**Gender**					
Female	150	140(93.33)	4(2.67)	6(4.00)	0.395
Male	285	273(95.79)	3(1.05)	9(3.16)	
**YEAR**					
2016	43	41(95.35)	0(0.00)	2(4.65)	**0.038**
2017	143	135(94.41)	4(2.80)	4(2.80)	
2018	139	133(95.68)	0(0.00)	6(4.32)	
2019	6	6(100.00)	0(0.00)	0(0.00)	
2020	34	29(85.29)	3(8.82)	2(5.88)	
2021	70	69(98.57)	0(0.00)	1(1.43)	

**Season**					
Rainy season	302	287(95.03)	4(1.32)	11(3.64)	0.738
Dry season	133	126(94.74)	3(2.26)	4(3.01)	

In each age group, there were more males the females that were burdened with TB. This notwithstanding, while the male and female burden of TB was comparable among the age group less than 15 years and those between 15 and 29 years, significantly higher infection burden were seen among males than females for the age groups 30-44 years, 45 to 59 years and ≥ 60 years, as shown in Figure 2.

Figure 2 Gender and variations in TB prevalence across age groups

The cumulative pulmonary tuberculosis burden within the period of the review was observed at the month September (15.7%). However, among the ~~male tuberculosis~~ cases were most frequently detected among suspects in May. Cumulatively, positivity to tuberculosis ~~d~~ burden was least observed in October and November. While among the males, tuberculosis positivity among suspects were least reported in November, October was the month with the least burden of tuberculosis positivity among females. The observed monthly variations in tuberculosis detection were statistically stable.

Comparable burden of tuberculosis was observed between male and female suspects in the months between June and September ($p>0.05$). On the contrary, from October and March significantly higher *Mycobacterium tuberculosis* burden was detected among the male suspects compared to females ($p<0.05$). Figure 3

Figure 3 Monthly variation in TB detection at the Ho Teaching Hospital, 2016 - 2021

There was a significant trend in tuberculosis prevalence over the period with a decline in tuberculosis prevalence from a peak in 2016 (17.1%) through to 2019 (4.6%) where there was a trough in tuberculosis prevalence. There was however a sharp increase in TB rate from 4.6% in 2019 to 11.5% in 2020 with slight decline in 2021 (9.7%) ($p\text{-value}_{(trend)}$ = 0.016). Though not significant, similar trend of tuberculosis infection was also seen among males and female when highest in tuberculosis was most prevalent in 2016 and least prevalent in 2019, as depicted in Figure 4.

Figure 4 Year on year trend of TB prevalence stratified by gender at the Ho Teaching Hospital

4.0 Discussion

The retrospective study conducted over six years at the Ho Teaching Hospital sheds light on the prevalence of laboratory-confirmed tuberculosis among the received presumptive cases. A total of 5128 presumptive TB cases were examined, with 48.89% being males and 51.11% being females. The overall prevalence of laboratory-confirmed tuberculosis stood at 10.76%, indicating a significant burden within the hospital's catchment area. Similar study by Arega, Menbere [9] in Addis Ababa and Boakye-Appiah, Steinmetz [10] in Komfo Anokye Teaching Hospital, Ghana reported a prevalence of 15.11% and 31.4% respectively. The high prevalence reported by Boakye-Appiah, Steinmetz [10] for TB could be due to the small amount of study population (376).

The prevalence was notably higher among males at 14.20% compared to females at 7.48%, revealing a distinct gender disparity in tuberculosis rates. This finding is supported by the male to female ratio of 2:1, signifying that males were at a significantly higher risk of being diagnosed

with TB compared to their female counterparts. Our study is consistent with studies by Gallant, Duvvuri [11], Lisdawati, Puspandari [12], Moyo, Ismail [13] and Sinshaw, Kebede [5].. In contrast to our study, being female was identified as an independent risk factor for RR-TB in a study by Arega, Menbere [9] and this may be related to socioeconomic factors, probably due to lack of control of financial resources at household levels and poor knowledge regarding tuberculosis.

Moreover, the study's adjusted odds ratio of 2.07 (95% CI: 1.72-2.49) further emphasizes the increased likelihood of males being burdened with TB compared to females, even after accounting for potential confounding variables. This gender-based disparity in tuberculosis prevalence suggests a possible underlying biological or behavioral vulnerability among males, underscoring the need for targeted interventions and awareness campaigns tailored to the specific challenges faced by this demographic.

In addition to gender-based differences, the burden of laboratory-confirmed tuberculosis exhibited substantial variations across different age groups. Notably, individuals below 15 years of age demonstrated the lowest prevalence, indicating a relatively lower susceptibility to tuberculosis within this cohort. On the other hand, the middle-aged group (30-45 years) exhibited the highest risk, with a prevalence rate of 13.35%. The adjusted odds ratios for the age groups 30-45 years and 46-60 years stood at 2.74 (95% CI: 1.74-4.31) and 2.58 (95% CI: 1.64-34.07) respectively, indicating a significantly elevated likelihood of TB occurrence compared to those under 15 years. This is consistent with a study by Kapata, Chanda-Kapata [14].

Furthermore, the study highlighted the association between climatic seasons and tuberculosis occurrence, indicating a significant link between the prevalence of tuberculosis and the climatic conditions in the area. The identification of tuberculosis cases was notably more frequent during the rainy season, accounting for 12.12% of cases, compared to 8.84% during the dry season. This

observation underscores the potential impact of environmental factors on the transmission dynamics of tuberculosis and emphasizes the need for targeted surveillance and preventive measures during specific climatic periods, especially during times of increased rainfall. These findings underscore the complex interplay of biological, demographic, and environmental factors in influencing the prevalence of tuberculosis. They emphasize the need for tailored public health interventions and comprehensive strategies aimed at addressing the specific vulnerabilities identified among different demographic groups and during specific climatic seasons. This comprehensive approach is crucial for mitigating the burden of tuberculosis and improving the overall health outcomes within the hospital's catchment area.

The findings from the retrospective study conducted at the Ho Teaching Hospital revealed several important patterns regarding the prevalence of newly diagnosed rifampicin-resistant Tuberculosis (RR-TB). The study found an overall prevalence rate of 3.45% for RR-TB, indicating a significant proportion of the tuberculosis cases in the region being resistant to this crucial antibiotic. Other studies in Ghana by Boakye-Appiah, Steinmetz [10] and Sylverken, Kwarteng [15] reported a Rifampicin resistance of 14.4% and 2.4% respectively.

Surprisingly, the prevalence of RR-TB showed a slight predominance among females compared to males, with rates of 4.00% and 3.16%, respectively, although the observed difference was not statistically significant ($p>0.05$). This suggests that gender might not be a significant determinant in the occurrence of RR-TB within this population.

Moreover, the study indicated that the age distribution of RR-TB cases did not show a stark variation, with comparable prevalence rates across different age groups. However, a notable observation was the relatively higher burden of resistance among the 45-59 age group, with a prevalence of 5.97%. Notably, no RR-TB cases were identified among patients below 15 years,

implying a potential age-related factor in the resistance pattern. This trend could suggest a certain level of vulnerability among middle-aged individuals, indicating a need for targeted interventions in this specific demographic group.

Interestingly, the study highlighted a significant fluctuation in the burden of newly diagnosed RR-TB over the six-year period under review ($p<0.05$). The prevalence of RR-TB exhibited an oscillating trend across the years, showing a pattern of inconsistency rather than a steady increase or decrease. This fluctuation was evident from the observed rates over the years, starting at 4.65% in 2016, followed by a decrease in 2017 to 2.80%, an increase in 2018 to 4.32%, a complete absence in 2019, a substantial increase in 2020 to 5.88%, and a subsequent decrease to 1.43% in 2021. These variations suggest the presence of potential contributing factors that might have influenced the dynamics of RR-TB prevalence during the study period.

Conversely, the study results indicated no significant association between climatic seasons and the occurrence of rifampicin resistance among the newly diagnosed tuberculosis cases ($p>0.05$). This finding suggests that the prevalence of RR-TB remained consistent across different seasons, implying that environmental factors, specifically those related to climate variations, might not be significant drivers of RR-TB prevalence within the study area.

The study provides critical insights into the prevalence of rifampicin-resistant Tuberculosis within the Ho Teaching Hospital catchment area, highlighting the need for further research to elucidate the underlying factors driving the observed trends. Additionally, the study underscores the importance of continuous monitoring and surveillance of RR-TB to implement targeted interventions and policies that can effectively combat the challenges posed by antibiotic resistance in tuberculosis management.

The results of this study, indicate a notable prevalence of tuberculosis cases among males across all age groups, suggesting a potential gender-based disparity in the burden of the disease. Surprisingly, the study reveals a relatively balanced distribution of tuberculosis between males and females in the age groups under 15 years and between 15 and 29 years. This balanced representation in the younger age cohorts may indicate a comparable vulnerability to tuberculosis infection in both genders, possibly owing to similar exposure patterns and physiological factors influencing susceptibility during these stages of life.

However, a significant deviation from this trend emerges in the subsequent age categories, 30-44 years, 45-59 years, and 60 years and above. In these age groups, the burden of tuberculosis demonstrates a strikingly higher incidence among males compared to females. This disparity suggests a potential interplay of multiple factors, including disparities in healthcare access, occupational hazards, behavioral patterns, and hormonal influences, which might contribute to the elevated vulnerability of males to tuberculosis within these older age brackets.

The observed gender-based differences in tuberculosis prevalence, particularly in the older age groups, point to the need for a more nuanced understanding of the social, biological, and environmental determinants that contribute to the unequal distribution of tuberculosis burden. Moreover, it emphasizes the importance of implementing targeted interventions tailored to address the specific risk factors and challenges faced by males in the older age cohorts, such as improved access to healthcare services, heightened awareness campaigns, and comprehensive screening programs, to mitigate the disproportionate impact of tuberculosis within these populations. Further research is warranted to explore the intricate interplay of these factors and their role in shaping the gender-based disparities in tuberculosis prevalence, ultimately facilitating the development of

more effective and tailored public health strategies aimed at curbing the tuberculosis burden across diverse demographic groups.

5.0 Conclusion

In conclusion, the retrospective study conducted at the Ho Teaching Hospital provides crucial insights into the prevalence of laboratory-confirmed tuberculosis and newly diagnosed RR-TB within its catchment area. The findings underscore significant gender disparities in tuberculosis prevalence, with males exhibiting a higher burden across all age groups, while females demonstrated a relatively balanced representation in the younger age cohorts. This highlights the need for targeted interventions and awareness campaigns tailored to address the specific challenges faced by males, especially in older age brackets, including improved healthcare access, awareness campaigns, and comprehensive screening programs. Furthermore, the study emphasizes the importance of continuous monitoring, surveillance, and further research to elucidate the underlying factors driving the observed trends, aiding the development of more effective public health strategies to mitigate the tuberculosis burden.

Conflicts of interest

The authors declared no conflicts of interest.

Authors' contributions

Conceptualisation: JGD, EA, SW, IB, PM and IA. Methodology: JGD, GN, AE, O-TA and DE. Project administration: JGD. Resources: JGD, AE, O-TA, SW, DE and IB. Investigation: JGD,

GN, and AE. Supervision: JGD, PM and IA. Data curation: IB, PM, GN, AE, O-TA and DE. Formal analysis: IB and AE. Writing-original draft: JGD, SW, EA, IA and IB. Writing-review and editing: JGD, EA and IA. All authors read and approved the final version of the manuscript.

Acknowledgments

The authors are grateful to the management of the Ho Teaching Hospital and the staff of the laboratory department for their support.

References

1. Natarajan, A., et al., *A systemic review on tuberculosis.* Indian Journal of Tuberculosis, 2020. **67**(3): p. 295-311.
2. Glaziou, P., K. Floyd, and M.C. Raviglione. *Global epidemiology of tuberculosis*. in *Seminars in respiratory and critical care medicine*. 2018. Thieme Medical Publishers.
3. Gautam, M., *Latent tuberculosis infection.* Peter DO Davies, MA, DM, FRCP, 2015: p. 139.
4. O'Garra, A., et al., *The immune response in tuberculosis.* Annual review of immunology, 2013. **31**: p. 475-527.
5. Sinshaw, W., et al., *Prevalence of tuberculosis, multidrug resistant tuberculosis and associated risk factors among smear negative presumptive pulmonary tuberculosis patients in Addis Ababa, Ethiopia.* BMC infectious diseases, 2019. **19**: p. 1-15.
6. Alene, K.A., Z.A. Wagaw, and A.C. Clements, *Mapping tuberculosis prevalence in Ethiopia: protocol for a geospatial meta-analysis.* BMJ open, 2020. **10**(5): p. e034704.
7. Delogu, G., M. Sali, and G. Fadda, *The biology of mycobacterium tuberculosis infection.* Mediterranean journal of hematology and infectious diseases, 2013. **5**(1).
8. GSS., *Population ans Housing Census, District Analytical Report, Ho Municipal. GSS (2010) 'Population and Housing Census, District Analytical Report, Ho Municipal'.* 2010.
9. Arega, B., F. Menbere, and Y. Getachew, *Prevalence of rifampicin resistant Mycobacterium tuberculosis among presumptive tuberculosis patients in selected governmental hospitals in Addis Ababa, Ethiopia.* BMC infectious diseases, 2019. **19**(1): p. 1-5.
10. Boakye-Appiah, J.K., et al., *High prevalence of multidrug-resistant tuberculosis among patients with rifampicin resistance using GeneXpert Mycobacterium tuberculosis/rifampicin in Ghana.* International journal of mycobacteriology, 2016. **5**(2): p. 226-230.
11. Gallant, V., V. Duvvuri, and M. McGuire, *Tuberculosis (TB): tuberculosis in Canada-summary 2015.* Canada Communicable Disease Report, 2017. **43**(3-4): p. 77.
12. Lisdawati, V., et al., *Molecular epidemiology study of Mycobacterium tuberculosis and its susceptibility to anti-tuberculosis drugs in Indonesia.* BMC infectious diseases, 2015. **15**: p. 1-8.
13. Moyo, S., et al., *Prevalence of bacteriologically confirmed pulmonary tuberculosis in South Africa, 2017–19: a multistage, cluster-based, cross-sectional survey.* The Lancet Infectious Diseases, 2022. **22**(8): p. 1172-1180.
14. Kapata, N., et al., *The prevalence of tuberculosis in Zambia: results from the first national TB prevalence survey, 2013–2014.* PLoS One, 2016. **11**(1): p. e0146392.

15. Sylverken, A.A., et al., *The burden of drug resistance tuberculosis in Ghana; results of the First National Survey*. PLoS One, 2021. **16**(6): p. e0252819.

Click here to access/download;Figure;Figure 1. Location of the Ho Teaching Hospital.docx

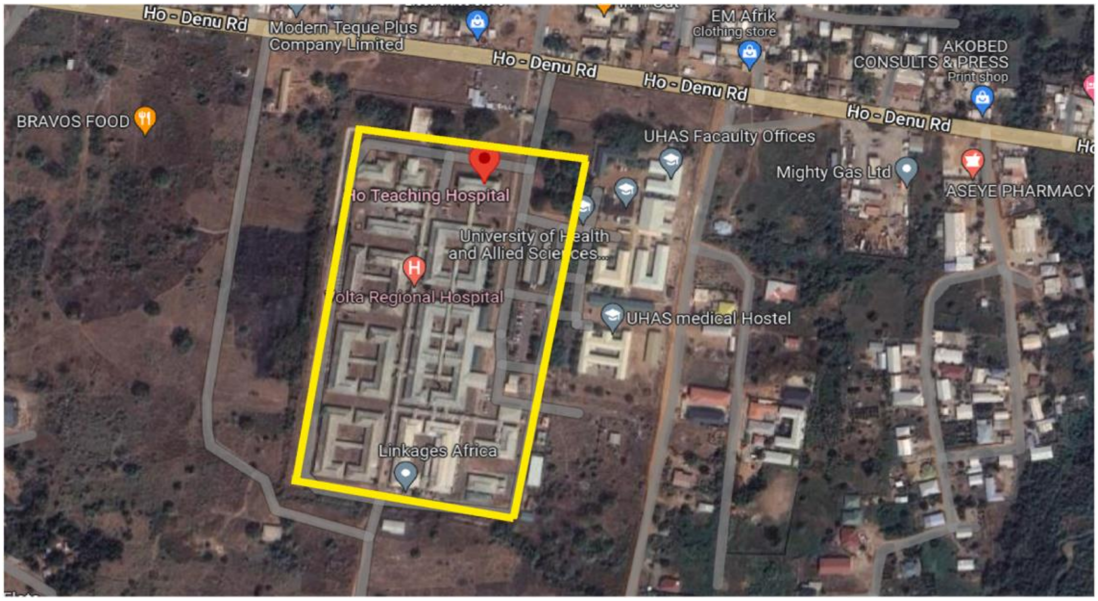

Figure

Click here to access/download;Figure;Figure 2 Gender and variations in TB prevalence.docx

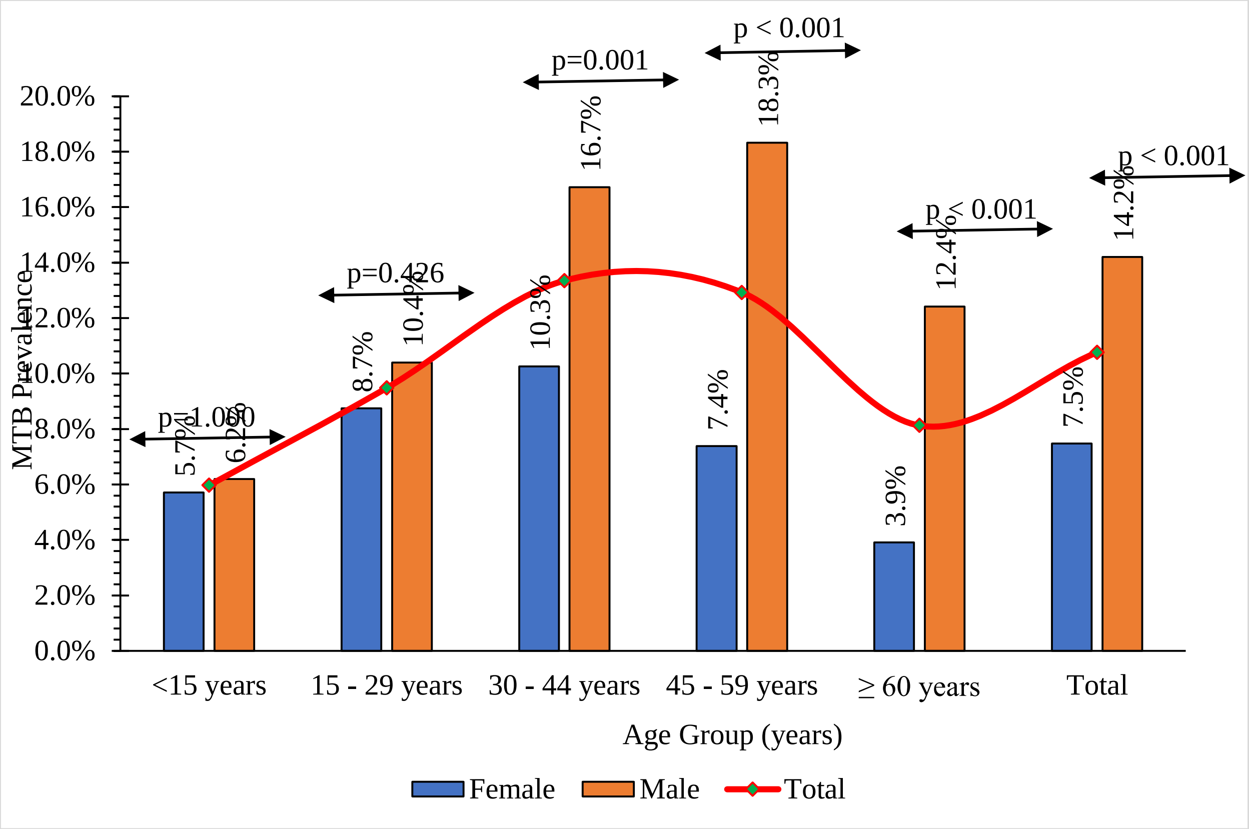

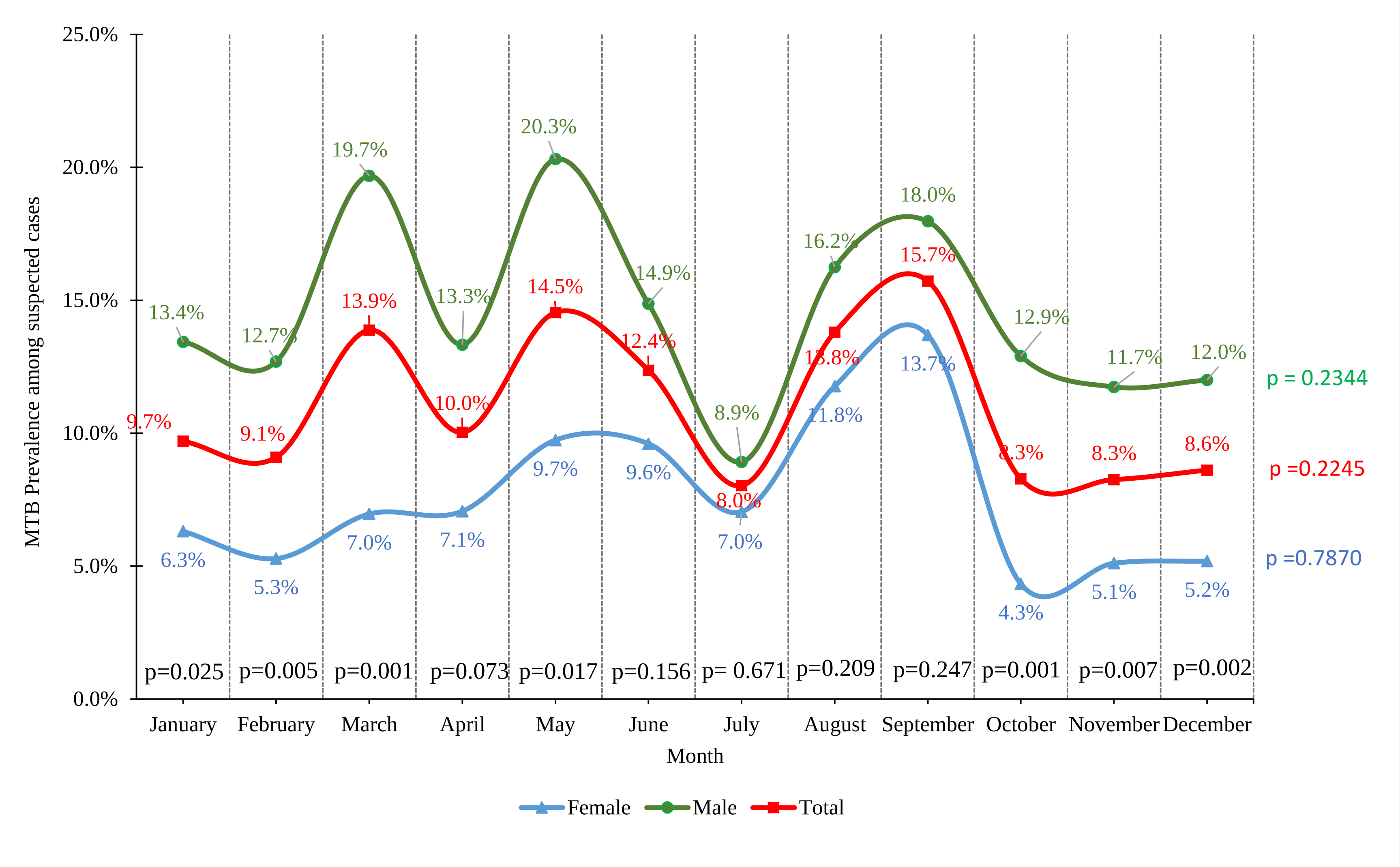

25.0%
20.0%
15.0%
10.0%
5.0%
0.0%
MTB Prevalence among suspected cases
13.4%
12.7%
19.7%
13.3%
20.3%
14.9%
8.9%
16.2%
18.0%
12.9%
11.7%
12.0%
9.7%
9.1%
13.9%
10.0%
14.5%
12.4%
8.0%
13.8%
15.7%
8.3%
8.3%
8.6%
6.3%
5.3%
7.0%
7.1%
9.7%
9.6%
7.0%
11.8%
13.7%
4.3%
5.1%
5.2%
p = 0.2344
p =0.2245
p =0.7870
p=0.025
p=0.005
p=0.001
p=0.073
p=0.017
p=0.156
p= 0.671
p=0.209
p=0.247
p=0.001
p=0.007
p=0.002
January
February
March
April
May
June
July
August
September
October
November
December
Month
Female
Male
Total

Figure

Click here to access/download;Figure;Figure 4 Year on year trend of TB prevalence.docx

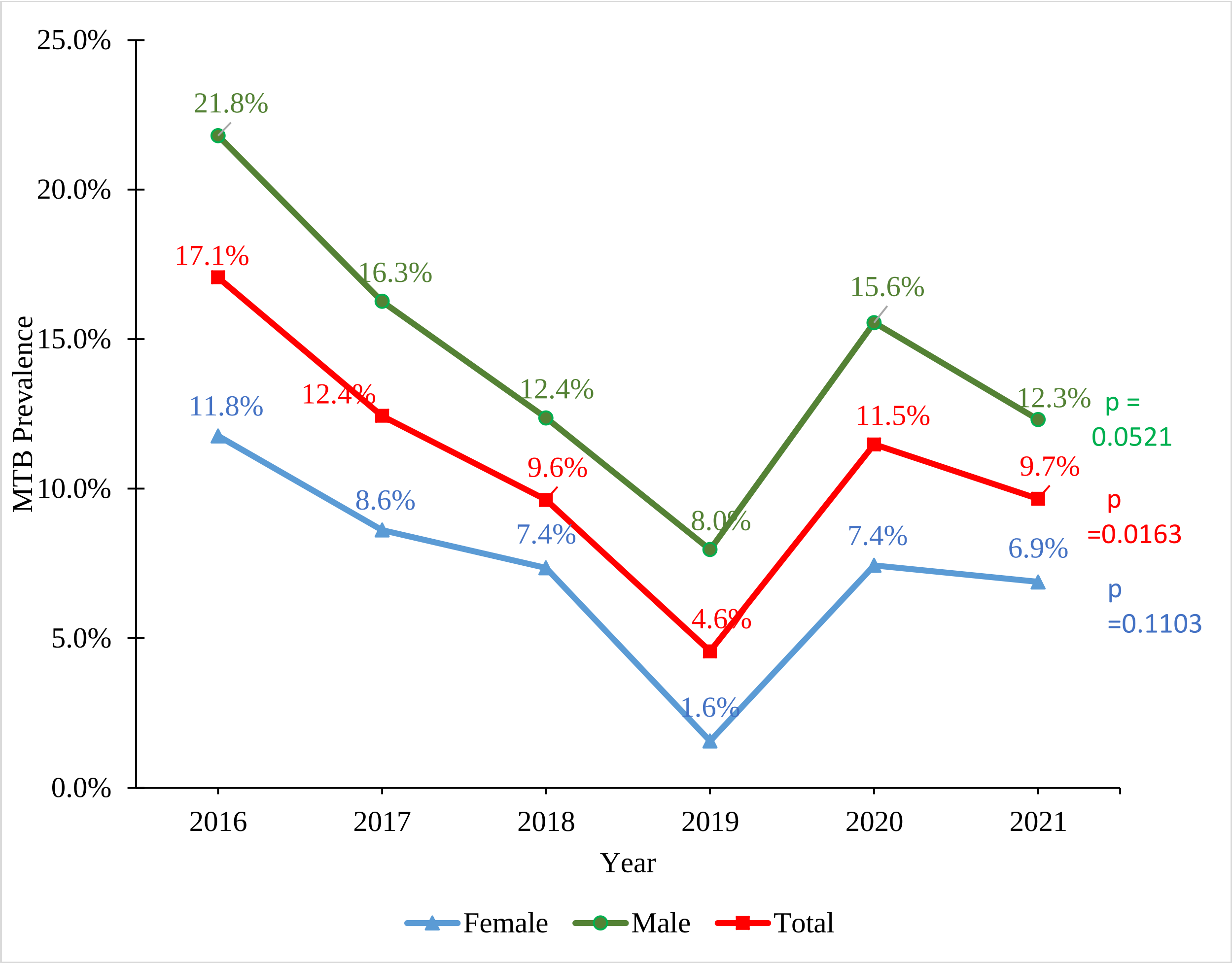

Supplement: Attachment — Submitted filename: PONE-D-23-36410_reviewer_Reviewed.pdf [file pone.0305161.s002.pdf]
