# Supplementary material for: Trends of Mycobacterium tuberculosis and rifampicin resistance at the Ho Teaching Hospital in Ghana
Source: PLoS One. 2024 Jun 10;19(6):e0305161. doi: 10.1371/journal.pone.0305161 (PMC11164350; doi:10.1371/journal.pone.0305161)
Supplement: Attachment — Submitted filename: Response to Reviewers.docx [file pone.0305161.s003.docx]

University of Health and Allied Sciences

PMB 31

Ho

January 25, 2024

The Editor

Plos One

Dear Editor,

RESPONSE TO REVIEWERS COMMENT

The authors of the manuscript “Trends of *Mycobacterium tuberculosis* and rifampicin resistance at the Ho Teaching Hospital in Ghana.”, with manuscript ID. PONE-D-23-36410 are grateful for your mail. We are equally thankful for the time you spent on the manuscript to enrich it for possible publication in your esteemed journal.

We write to respond to your comments as follows:

**Reviewer #1:**

**Comment**: “All comments have been incorporated in the marked manuscript.

For example, the introduction is silent on the subject matter which was supposed to have been studied. For example, there are several studies on Rifampicin resistance in Ghana but the authors rarely mentioned any of these”.

**Authors’ response**: All corrections have been made as suggested in the manuscript. However, the authors wish to clarify that, the months under the result section are cumulative over the six-year period. (lines 574-580)

**Reviewer#2:**

1. Title
**Reviewer comment**: “The title should be revised as “Trends of Mycobacterium tuberculosis and rifampicin resistance……”

**Authors’ response:** The title has been revised to “Trends of *Mycobacterium tuberculosis* and rifampicin resistance at the Ho Teaching Hospital in Ghana”. See lines 1 and 2.

2. Abstract section
**Reviewer comment:** “a. Lacks research gaps”

**Authors’ response:** The abstract has been revised to include the research gap. See lines 36-37.

**Reviewer comment:** “b. Its results were not support with statistical values and mix-up with recommendations”

**Authors’ response:** Authors have added statistical values (Lines 51 and 78) and removed recommendations.
**Reviewer comment:** “c. Its conclusion is not well stated”

**Authors’ response:** The conclusion has been restated. See lines 82-84.

**Reviewer comment** 3. Introduction
**Reviewer comment**: “a. Need a through language edition”

**Authors response:** Language editing was done as suggested.
**Reviewer comment** b “Inconsistence citation (line 96)”

**Authors’ response:** “All comments have been addressed.

4. Method section
**Reviewer comment**: “a. Study area description lacks coherence”

**Authors’ response:** This section has been rephrased to improve coherence.
**Reviewer comment:** “b. No criteria for inclusion/exclusion of data type/participants”

**Authors’ response:** The inclusion and exclusion criteria section for data type/participants have been added. See 239-242

**Reviewer comment:** “c. Data collection not well described; what variables were collected”

**Authors’ response:** Variables obtained for the study have been added to the data collection section. See line 361-367.

**Reviewer comment:** “d. Variables (outcome and independents variables) are missing”

**Authors response:** Variables (outcome and independents variables) session have been added. See line 361-367.

5. Results
**Reviewer comment** a. “This section should be reanalysis; the way you wrote AOR? Not to the standard”

**Authors response**: Authors are unable to work on this comment due to a lack of clarity on what is expected from us. However, the meaning of the abbreviations used in the table has been added as a footnote. See Table 1

6. Discussion
**Reviewer comment:** “a. You reviewed very few literatures for the discussion. Look at line number 253 to 306, you didn’t use any literature”.

“b. Overall you use 15 paper to prepare this manuscript”
**Authors response:** Comparative analysis has been done and more literatures cited.

7. Conclusion
**Reviewer comment:** “a. Your conclusion should be based on major findings; which is missed in this case”.

**Authors’ response:** Authors have revised study conclusions. See line 1010-1015

**Reviewer #3:**

**Reviewer comment:** “The manuscript is well written and method used appropriate to generate the data presented and analyzed. Although there is a concern on the results presenting data on rifampicin susceptibility testing (table as sensitive-Intermediate-Resistant) in table 2 (Table 2 Prevalence and risk factors of newly diagnosed rifampicin resistant tuberculosis among suspected 174 cases at the Ho Teaching Hospital between 2016 and 2021). The method used talked of results of Gene Xpert method and not the cultural method”.

**Authors response:** The Gene Xpert machine used reports rifampicin susceptibility results as “Susceptible”, “Indeterminate”, or “Resistant” where Indeterminate means neither “Susceptible” nor “Resistant” which could be due to insufficient bacterial load.

**Reviewer comment:** “There is also a limitation using this retrospective method which did not enable the authors to correlate the laboratory findings to the clinical presentations.”

**Authors response:** Suggested limitation of study has been added. See lines 1031- 1033

**Reviewer comment:** “In conclusion the study site was appropriate to determine the epidemiological trends given its high turn around and the number of suspicious of cases of M. tuberculosis per year. The data generated well appropriately analyzed and the conclusions are adequate to the study design”.

**Authors’ response:** Thank you.

Thank you.

…Signed……..

John Gameli Deku

(Lead and corresponding author)
