# Supplementary material for: Trends of Mycobacterium tuberculosis and rifampicin resistance at the Ho Teaching Hospital in Ghana
Source: PLoS One. 2024 Jun 10;19(6):e0305161. doi: 10.1371/journal.pone.0305161 (PMC11164350; doi:10.1371/journal.pone.0305161)
Supplement: Attachment — Submitted filename: Response to Reviewers_17052024.docx [file pone.0305161.s004.docx]

University of Health and Allied Sciences

PMB 31

Ho

May 17, 2024

The Editor-in-Chief

Plos One

Dear Editor-in-Chief,

RESPONSE TO COMMENTS

The authors of the manuscript “Trends of *Mycobacterium tuberculosis* and rifampicin resistance at the Ho Teaching Hospital in Ghana.”, with manuscript ID. PONE-D-23-36410R2 are grateful for your mail.

We wish to respond to the comments as follows:

**Comment**: “Please review your reference list to ensure that it is complete and correct.”

**Response**: The reference list has been reviewed.

**Comment**: “If you have cited papers that have been retracted, please include the rationale for doing so in the manuscript text, or remove these references and replace them with relevant current references.”

**Response**: No cited paper was retracted.

**Comment**: “Any changes to the reference list should be mentioned in the rebuttal letter that accompanies your revised manuscript.”

**Response**: There has been no change to the reference list.

**Comment**: “If you need to cite a retracted article, indicate the article’s retracted status in the References list and also include a citation and full reference for the retraction notice.”

**Response**: No retracted article has been cited.

Thank you.

…Signed……..

John Gameli Deku

(Lead and corresponding author)
